# Evidence Suggesting That Alzheimer’s Disease May Be a Transmissible Disorder

**DOI:** 10.3390/ijms26020508

**Published:** 2025-01-09

**Authors:** Genevieve Saw, Ling-Xiao Yi, Eng King Tan, Zhi Dong Zhou

**Affiliations:** 1 National Neuroscience Institute, 11 Jalan Tan Tock Seng, Singapore 308433, Singapore; genevievesaw@gmail.com (G.S.); ylxiao9435@hotmail.com (L.-X.Y.); 2 Department of Neurology, Singapore General Hospital, Outram Road, Singapore 169608, Singapore; 3 Signature Research Program in Neuroscience and Behavioral Disorders, Duke-NUS Medical School, 8 College Road, Singapore 169857, Singapore

**Keywords:** Alzheimer’s disease, transmissible disease, protein aggregation, neurodegeneration, pathogenesis, therapeutic strategies

## Abstract

Alzheimer’s disease (AD) is characterised by progressive neurodegeneration with the formation of amyloid beta (Aβ) plaques and neurofibrillary tau tangles in the brain parenchyma. The causes of AD have been attributed to a combination of age-related changes within the brain as well as genetic, environmental and lifestyle factors. However, a recent study by Banerjee et al. highlights the possibility that AD may be a transmissible disease and that iatrogenic AD could be environmentally acquired, similar to iatrogenic Creutzfeldt–Jakob disease (iCJD). The study reports that contaminated Aβ in cadaver-derived pituitary growth hormone (c-hGH) therapy, which patients received during childhood inoculation, may accidentally transmit into their brains, triggering neurodegeneration and AD onset in older age. Furthermore, corroborating evidence from various animal model studies and human case reports suggests that AD can be potentially transmissible.

Alzheimer’s disease (AD) is the most common progressive neurodegenerative disease that impairs the memory and cognitive ability of affected subjects [1]. AD is also the most prevalent form of dementia, accounting for around two-thirds of total dementia cases in patients aged 65 and above [2]. The pathological hallmarks of AD consist of the accumulation of amyloid beta (Aβ) plaques and formation of neurofibrillary tangles of tau protein in the brain parenchyma [3]. The exact pathogenesis of AD is still unclear, though multiple factors are believed to contribute to it, including aging, genetic predisposition, and environmental and lifestyle factors [4,5]. There is evidence to suggest that accumulation of Aβ peptides (typically present as a fibrillar morphology and an ordered “cross-β” assembly) is the pathogenic trigger for pathological cascade, contributing to the spreading of tau protein, brain structural changes and neurodegeneration [6,7]. It was reported previously that Aβ peptides had been transmitted in young adult patients who died of iatrogenic Creutzfeldt–Jakob disease (iCJD) after receiving treatment during their childhood with cadaver-derived pituitary growth hormone (c-hGH) contaminated with both CJD prions and Aβ seeds. However, recent findings demonstrate that patients who receive Aβ-contaminated c-hGH treatment may develop AD rather than iCJD [8]. These findings suggest that Aβ seeds can penetrate the blood–brain barrier (BBB) and enter patients’ brains, triggering pathological changes and AD onset, indicating that AD may be another transmissible human neurodegenerative disorder similar to prion diseases.

Prion diseases are caused by misfolding and aggregation of cellular prion proteins (PrP^C^) which are glycosylphosphatidylinositol (GPI)-anchored proteins most prevalently localized in the neuron outer membrane [9]. Prion diseases are transmissible and infectious neurodegenerative diseases involving a protein-only infectious agent of misfolded prion proteins that is able to propagate between individuals [10]. In fact, prion proteins are considered a subclass of amyloids, wherein prion proteins aggregate to become self-perpetuating and infectious [11]. The recent study by Banerjee et al. highlights a similarity between the transmission of Aβ peptides and prion proteins, showing that the pathogenic Aβ peptides can spread between individuals to induce neurodegeneration in a similar manner to that of prion proteins [12]. In the study, Banerjee et al. studied eight AD patients who had received a c-hGH injection prepared with a method known as the Wilhelmi or Hartree-modified Wilhelmi preparation (HWP), which has been implicated in all cases of iatrogenic CJD within the United Kingdom [13,14]. Five of these eight patients were found to have symptoms of early-onset dementia, including progressive cognitive impairments affecting the performance of their daily activities, while the remaining three patients had symptoms of mild cognitive impairment predominantly affecting their personality and behaviour [8]. Of the five patients exhibiting symptoms of early-onset dementia, three had already been clinically diagnosed with AD prior to this study.

The notion that AD could be a new transmissible disease is supported by findings that there were measurable quantities of Aβ species and tau protein in archived batches of c-hGH prepared via the HWP method [15]. Experiments in animal models have shown that misfolded Aβ can act as a “seed” capable of inducing Aβ pathology in the living brain and that this pattern of protein transmission is reminiscent of prion infectivity [16]. Furthermore, Aβ plaque deposition was found in the brains of mice inoculated intracerebrally with human brain homogenates from AD patients, but not in those inoculated with homogenates from healthy human subjects [15]. The intracerebral injection of AD patient brain homogenate with amyloid plaques led to cerebral amyloidosis and neurodegeneration in a primate model [17]. The inoculation of human AD brain extracts into the cortex of primates led to accumulation of Aβ and tau pathologies and this was associated with cognitive deficits, suggesting transmission of both Aβ and tau pathologies [18]. The transmission of tau pathology was also validated in a mice model, as the injection of brain extracts from AD mice with the tau P301S mutation into healthy mouse brains led to AD tau pathology in the injected brains [19]. Except for the oral route, administration of AD patient brain samples with Aβ seeds via peripheral routes, including intra-venous, intra-peritoneal, intra-muscular and even dropping brain homogenate with Aβ seeds into eyes, can induce mice cerebral amyloid pathology with Aβ accumulation [20]. These findings suggest that AD can be a transmissible disorder.

There are limitations in the study by Banerjee et al., such as small sample size of AD subjects, lack of sufficient tau and Aβ biomarkers, atypical AD patient phenotypes and a limited amount of contaminated Aβ-40 in archived HWP c-hGH samples [21,22]. However, accumulative evidence from other human case reports suggests that AD is a transmissible disease [23,24,25,26,27,28,29,30,31,32,33,34,35]. Besides the study by Banerjee et al., multiple cases of transmission of Aβ amyloid and tauopathy from injection of human cadaver-derived growth hormone have been reported previously [23,34,35]. The presence of substantial levels of pathogenic Aβ40, Aβ42 and tau proteins has been identified in archived c-hGH vials [25]. It has been found that the intracellular Aβ oligomers trigger the initial phase of Aβ seeding [36], which can transfer via direct neuronal connections, contributing to neurodegeneration and disease progression [30]. The deposition of pathogenic Aβ in the brain can be a long-term pathological process [37]. The mean incubation period for the development of detectable and typical pathogenic Aβ deposits in human brains would be longer than 18 years from the first exposure to Aβ seeds [15,37]. Moreover, evidence indicates that tau pathology could also be transmissible in humans, leading to progression of neurofibrillary pathology [26,27,31,37]. However, brain Aβ can accelerate the spread of tau protein through neuronal communication and connections [26]. There is also evidence showing the transmission of Aβ and tau pathologies to host brains due to contaminated dural mater transplantation [26,27,28,31,32,37], and there is data linking dura mater grafting with subpial Aβ deposition and amyloid angiopathy [31]. For example, a 34-year-old male with intracerebral haemorrhages and severe cerebral amyloid angiopathy (CAA) was found to have received cardiac surgery with a cadaveric dura mater patch three decades ago, suggesting the transmission of pathological Aβ seeds from the periphery into the patient brain [35]. Moreover, Aβ amyloid can also be transmitted via other neurosurgical procedures [24]. Four patients who had undergone neurosurgical operations at young ages were observed to develop intracerebral haemorrhages with serious CAA three decades later [24]. However, no pathogenic AD gene mutations have been identified in these subjects [24]. This finding can cause a public health concern, if Aβ and tau pathologies can be transmitted during neurosurgical operations [35,38,39].

Pathogenic Aβ and tau seeds are believed to account for AD transmissibility; however, recent studies suggest that infectious agents, such as viruses, may also contribute to it. The syphilis caused by Treponema pallidum can lead to dementia [40]. However, epidemiological investigations indicate that human subjects vaccinated against shingles have a significantly decreased risk of dementia prevalence [41]. The herpes simplex virus (HSV) and other infectious microorganisms can inhabit elderly individuals’ brain functions and impair memory and learning [42]. The borrelia burgdorferi could aggregate and co-localize with Aβ amyloid and hyperphosphorylated tau protein in Lyme disease [42]. Rapid AD-like plaque and neurofibrillary tangle formations have been identified after HSV or bacterial infection in mice [43]. In a similar manner to prion protein, the transmissible microbes, such as HSV, promote Aβ oligomer formation and tau protein hyperphosphorylation [42]. Furthermore, the microbe infections can cause Aβ deposition and up-regulation of prion protein, which leads to high affinity interaction between prion protein and Aβ oligomers, followed by Fyn signaling pathway activation and neurodegeneration [44]. The treatable and transmissible AD neurodegeneration and progression triggered by microorganism infection warrants further study and investigation.

There is a need for additional studies to corroborate current observations [16]. The potential for Aβ and tau transmission during routine neurosurgical procedures or other medical interventions raises significant public health concerns. Investigating approaches to mitigate the effects of transmitted pathogenic protein seeds through targeted therapies could be a promising direction. Novel sterilization protocols, surgical materials and strategies to minimize the risks of inadvertent transmission may prevent the transmissibility of deleterious protein seeds during medical practice. New approaches may be developed to enhance the clearance of misfolded proteins, block their propagation or fortify the BBB to prevent entry into the brain. Large scale epidemiological studies to investigate AD prevalence in populations with or without childhood c-hGH inoculation, graft transplantation and surgeries would be useful. However, as the investigation on human subjects involves detailed medical history studies, it could be a challenge to provide large-scale epidemiological evidence of a cause-and-effect relationship in Aβ and tau protein seed transmission-induced AD. It will be interesting to determine whether the injection of a tiny amount of contaminated Aβ and tau protein seeds can lead to the AD phenotype in primate and mouse models. The time duration between injection of Aβ and tau protein seeds and onset of the AD phenotype is also an important issue to be studied. Furthermore, the processing of Aβ has not been fully understood. It will be important to demonstrate how peripheral inoculated Aβ and tau protein seeds can be transported to the brain, penetrate the BBB and enter brain neurons. Understanding how these misfolded proteins are transported from peripheral inoculation sites to neurons may provide critical insights into the early stages of AD pathogenesis. The abnormal aggregated Aβ in the extracellular space is toxic to the neuron. Nevertheless, numerous studies have indicated that the intraneuronal accumulation of Aβ is also involved in the pathogenesis of AD [45,46,47]. Studies should aim to clarify how peripheral protein seeds contribute to this process and determine whether interventions targeting intraneuronal Aβ can slow or prevent neurodegeneration. These studies could provide a clearer understanding of the potential iatrogenic transmission pathways and their contribution to overall AD risk. Currently, few AD biomarkers are available to monitor disease progression, especially at the early pre-clinical stage. Future development of sensitive biomarkers to detect low levels of Aβ and tau seeds in biological samples will enable early diagnosis and detection of transmissible AD cases. This would also aid in monitoring the progression of AD pathology in experimental and clinical studies.

In summary, current available evidence from in vitro, animal and human case reports support the notion that Aβ and tau pathologies can be transmitted in vivo, similar to what has been observed in prion diseases, suggesting that, at least in some instances, AD may potentially be a transmissible disorder. The impacts of the notion that AD is transmissible would be profound, spanning medical research, clinical practice, public health policies and the societal perception of the disease. The potential for transmission of pathogenic AD proteins through medical procedures, blood transfusions or contaminated surgical instruments could prompt a reevaluation of sterilization techniques, especially for neurosurgical tools and procedures involving brain tissue. Furthermore, guidelines for tissue handling, organ donation and cerebrospinal fluid (CSF) exposure would be revised to minimize the risk. At the public health level, the discovery could trigger awareness campaigns to educate healthcare providers and the public about the modes of AD transmission. Society may view AD not just as a degenerative, age-related condition but also as one with external risk factors that can be mitigated. This shift could further incentivize early intervention and preventive care, reshaping the global strategy to combat AD.

## Abbreviations

Aβ: amyloid beta, AD: Alzheimer’s disease, BBB: blood–brain barrier, c-hGH: cadaver-derived pituitary growth hormone, CAA: cerebral amyloid angiopathy, CSF: cerebrospinal fluid, GPI: glycosylphosphatidylinositol, HWP: Hartree-modified Wilhelmi preparation, iCJD: iatrogenic Creutzfeldt–Jakob disease, PrPC: prion proteins.
